# Colloidal Particles for Pickering Emulsion Stabilization Prepared via Antisolvent Precipitation of Lignin-Rich Cocoa Shell Extract

**DOI:** 10.3390/foods10020371

**Published:** 2021-02-09

**Authors:** Holly Cuthill, Carole Elleman, Thomas Curwen, Bettina Wolf

**Affiliations:** 1 Division of Food, Nutrition and Dietetics, Sutton Bonington Campus, The University of Nottingham, Loughborough LE12 5RD, UK; holly.cuthill@hotmail.co.uk; 2 The Reading Science Centre, Whiteknights Campus, Mondelēz International, Reading Scientific Services Ltd., Pepper Lane, Reading, Berkshire RG6 6LA, UK; carole.elleman@rssl.com (C.E.); Thomas.Curwen@mdlz.com (T.C.); 3 School of Chemical Engineering, University of Birmingham, Birmingham B15 2TT, UK

**Keywords:** lignin, antisolvent precipitation, colloidal lignin particles, oil-in-water emulsions, surface tension, Pickering emulsions

## Abstract

This study concerns the preparation and functionality testing of a new class of Pickering particles for food emulsion stabilization: colloidal lignin-rich particles (CLRPs) derived from ethanol-soluble extract of cocoa shell. A further goal was to achieve Pickering functionality without the need to add co-emulsifying surfactants during emulsion processing. Cocoa shell is a co-product of the food manufacturing industry. As such it is anticipated that the particles would be accepted as a natural food ingredient, provided no harmful solvents are used in any step of their processing. The cocoa shell particles were milled, dispersed in water and exposed to 250 °C for 1 h in a stainless-steel tubular reactor followed by ethanol extraction to obtain a lignin-rich extract (46% (*w*/*w*) lignin with the remainder predominantly lipids). CLRPs were then fabricated by the precipitation of ethanol-dissolved extract into water (antisolvent). By employing an agitated process and droplet dosing into a non-agitated process, four particle suspensions of a range of submicron diameters were obtained. All particle suspensions contained the same mass fraction of extract and were surface active, with surface tension decreasing with increasing particle size. The smallest particles were obtained when lipids were removed from the extract prior to particle processing. In contrast to the other four particle suspensions, this one failed to stabilize a 10% (*w*/*w*) sunflower oil-in-water emulsion. We hypothesize that the phospholipids indigenously present in these CLRP formulations are a critical component for Pickering functionality. It can be concluded that we have successfully introduced a new class of Pickering particles, fabricated from an industry co-product and anticipated to be food grade.

## 1. Introduction

Particles for the stabilization of emulsion interfaces are widely researched because particle stabilized or so-called Pickering emulsions [1] tend to be extremely stable against Ostwald ripening and coalescence [2]. This is related to the fact that particle adsorption at emulsion interfaces is practically irreversible, once adsorption has occurred in the first place [3]. Particle stabilized emulsion interfaces have also been reported to be robust against changes in solvent conditions post emulsion processing, such as pH and ionic strength. This can be a very useful property for manufactured emulsions, for example for food emulsions where pH may be reduced post emulsion processing to protect the end product against microbial spoilage. Indeed, several types of food-grade microparticles for emulsion stabilization have been described in literature. These include fat crystals [4], particles formed from sodium stearoyl lactylate [5], octenyl succinic anhydride (OSA) modified starch granules [6], ethyl cellulose [7], colloidal zein particles [8] as well as ‘lumpy’ shellac/ethyl cellulose rods with incorporated microparticles [9], cocoa particles [10] and spent coffee particles [11]. Further food-grade Pickering particles are mentioned in a number of review papers [12,13,14].

In the same year as Gould et al. [11] highlighted the role of lignin contributing to the Pickering functionality of spent coffee particles, Stewart et al. [15] reported on wood extract-based lignin microparticles but only hinted at functionality as food ingredient. Due to the size of the coffee particles, resulting emulsion droplets were larger than usually found in food emulsions, which is between 1 and 10 µm or smaller, such as salad dressings or culinary sauces. The source of the lignin employed in [15] on the other hand was nonfood, which may be undesired in food formulations. Therefore, we designed this study based on cocoa shell, containing 11.5–17% lignin [16], as the feedstock to close the gap between a food-grade lignin source and a sufficiently small particle size to be relevant to Pickering stabilization of food emulsions.

Lignin is a complex polymer of aromatic alcohols and found in the plant cell wall of terrestrial plants [17]. After cellulose, it is the second most abundant natural polymer [18]. It is partially hydrophobic and therefore a candidate natural material for Pickering particle formulation. This requires the particles to be wetted by both emulsion phases with one phase wetting preferentially over the other. The less wetted emulsion phase then tends to form the droplet phase, i.e., predominantly hydrophilic particles will preferably stabilize oil-in-water emulsions. Particle hydrophobicity can be assessed through the contact angle of a water droplet placed onto a flat surface of the particle material and surrounded by the nonpolar emulsion phase, or air. Wetting or nonwetting of the water droplet identifies the particles as hydrophilic and hydrophobic, respectively, with a contact angle of less and larger than 90° measured through the water phase.

In this study, cocoa shell particles were milled, dispersed in water and exposed to 250 °C for 1 h in a stainless-steel tubular reactor to maximize the yield of ethanol-soluble extract. During this hydrothermal treatment the lignin component of the cell wall material liquefies and migrates to the surface of the particles and the process medium [19]. Upon cooling, the hydrophobic lignin, and other hydrophobic feedstock components dissolved during the hydrothermal treatment, coalesce in the form of droplets onto the particle surface. A good lignin solvent such as ethanol [20] can then be utilized to obtain a lignin-rich extract for colloidal particle precipitation into water as antisolvent. Keeping extract concentration below 10 mg extract/mL ethanol was expected to lead to monodisperse colloidal particle dispersions [21]. Finally, particle properties relevant to emulsion stabilization were analyzed, including surface activity, size, morphology and surface charge. Oil-in-water emulsions were prepared, and droplet size evolution monitored for seven days. This study was undertaken to demonstrate the (partial) conversion of a food-grade lignin-rich feedstock into food-grade Pickering particles, but also to assess the impact of particle processing parameters and material properties on the resulting emulsion properties.

## 2. Materials and Methods

### 2.1. Materials

Cocoa shell was obtained as a co-product from the chocolate making process (Mondelēz International). The shells were milled to a powder using a hammer mill (LM 3100, Perten Instruments, Hägersten, Sweden). Particle size analysis in aqueous suspension, using the same method as described below for emulsions, revealed a volume based mean diameter of 69.8 ± 0.4 µm (d_4,3_). Reported as a measure of the fine fraction, the volume-based diameter below which 10 % of the particles are found, was 9.5 ± 0.1 µm (d_10,3_).

Acetyl bromide (Sigma-Aldrich, Dorset, UK), glacial acetic acid (Fisher Scientific, Loughborough, UK), hydroxylamine hydrochloride (NH_2_OH·HCl), sodium hydroxide (NaOH) and low sulfonate Kraft lignin (Sigma-Aldrich, Dorset, UK) were acquired as chemicals applied in the acetyl bromide soluble lignin (ABSL) assay to quantify the lignin content of the milled cocoa shell powder and its ethanol-soluble extract prepared with absolute ethanol (Fisher Scientific, Loughborough, UK). Where required Purite water was used. To investigate the impact of lipids on particle properties and performance, hexane (Fisher Scientific, Loughborough, UK) was used to remove co-extracted lipids. Sodium azide (Sigma-Aldrich, Dorset, UK) was added as an antimicrobial agent to all aqueous colloidal particle dispersions (0.01 % (*w*/*w*)). Sunflower oil (Sainsbury’s, London, UK) was treated with magnesium silicate (Sigma-Aldrich, Dorset, UK) to remove naturally present surface-active components. A constant interfacial tension of 27.1 ± 2.9 mN·m^−1^, measured with the pendant drop method introduced below, served as confirmation of a successful treatment. The treated sunflower oil, in the following simply referred to as sunflower oil, was used in contact angle measurements, interfacial tension measurements and as the oil phase in emulsion processing. All concentrations are provided on a *w*/*w* basis unless otherwise stated.

### 2.2. Acetyl Bromide Soluble Lignin Assay

The acetyl bromide soluble lignin (ABSL) method, adapted from literature [22], was used to quantify the lignin content of the cocoa shell powder and its ethanol-soluble extract as follows. 100 mg of sample was placed in a glass tube with 4 mL of solvent; 1:3 acetyl bromide: acetic acid. The mixture was placed in a shaking incubator at 50 °C for 2 h to solubilize the lignin. After cooling for 5 min in cold water (≈8 °C), 0.5 mL was added to a tube containing 7.5 mL of acetic acid, 1.5 mL of 0.3 M NaOH and 0.5 mL of 0.5 M NH_2_OH·HCl and vortexed until homogenous. The lignin content was then determined through UV absorption spectroscopy (UV-Vis Varian Cary 5D, Agilent Technologies, Santa Clara, CA, USA) at 280 nm based on calibration with Sigma Kraft lignin. Results reported correspond to the average of repeated analysis (2×) of the duplicate prepared samples.

### 2.3. Microscopy Methods

Scanning electron microscopy (SEM) was applied to image the surface morphology of the cocoa particles at various stages of the process. Dried cocoa particles were placed on SEM stubs with carbotape (TAAB, Berkshire, Japan), dried under vacuum and coated using a gold sputter (Leica SCD 0005, Leica Microsystems, Milton Keynes, UK). Images were then taken on a SEM (JSM 6060LV, JEOL, Tokyo, Japan) at the magnification of ×2000 using a 10 kV beam of electrons. Transmission electron microscopy (TEM) was applied to image the microstructure of produced colloidal particles as follows. The colloidal particle dispersions were pipetted onto holey carbon film grids on 300 mesh copper and dried for 2 days at 20 °C. Images were then taken on a TEM (Technai, FEI Thermo Fisher Scientific, Loughborough, UK) at 34,000, 60,000, 87,000 and 105,000× magnification to produce images with scale bars ranging from 0.2 to 2 µm. Finally, the microstructure of emulsions processed with the colloidal particle dispersions was visualized utilizing bright field microscopy (EVOS f1, AMG, Washington, DC, USA). A drop of undiluted emulsion sample was placed onto a glass microscope slide using a spatula and, without using a coverslip, images were taken at 40× magnification. Microstructure was visualized 1 day and 7 days after emulsion preparation in conjunction with droplet size analysis to assess droplet size stability.

### 2.4. Fourier Transform Infrared Spectroscopy (FT-IR)

The chemical composition of the extracted material prior to and post hexane treatment was fingerprinted via FT-IR spectroscopy. Spectra were collected utilizing a FT-IR (Tensor 27, Bruker, MA, USA) fitted with a diamond lens attenuated total reflectance module at room temperature (20 °C). The individual spectra were normalized using the mean and standard deviation for each data set:

(1) $$\mathrm{Normalized}\;\mathrm{absorbance}\;=\;\frac{\mathrm{Absorbance}\;\mathrm{at}\;\mathrm{each}\;\mathrm{wavenumber}-\mathrm{Mean}\;\mathrm{absorbance}\;\mathrm{of}\;\mathrm{data}\;\mathrm{set}}{\mathrm{Standard}\;\mathrm{deviation}\;\mathrm{of}\;\mathrm{the}\;\mathrm{mean}\;\mathrm{for}\;\mathrm{the}\;\mathrm{data}\;\mathrm{set}}$$

The spectra shown are the average of 3 normalized sets for each sample in the fingerprint region for lignin 800–2000 cm^−1^ [23]. The data was assessed qualitatively by comparing peaks to literature.

### 2.5. Lignin Extraction

Lignin was extracted from the cocoa shell to obtain a lignin-rich material for producing colloidal particles. The two-step process applied comprised a hydrothermal treatment followed by ethanol extraction of the treated and then dried biomass. For hydrothermal treatment, 8 g of powder was suspended in 30 mL of water and sealed into stainless steel tube reactors (3 mm diameter, 17 mm long). Loaded reactors were held at 250 °C in a muffle furnace (Carbolite, Hope, UK) for 1 h followed by cooling for 5 min by submerging the reactors into cold water (≈8 °C). Treatment temperature was selected to be well above the liquefaction temperature of lignin, reported to be between 100 and 170 °C [24], and optimized to show on SEM micrographs the largest quantity of droplets on the surface of the biomass, a method established with ground coffee waste [11].

The processed biomass was dried at 40 °C for 4 days in a vacuum oven (Weiss Gallenkamp, Leicestershire, UK) to a final moisture content of 2.28 ± 0.04% (wet basis). Subsequently, ethanol extraction was carried out by agitating the dried solids with ethanol at a ratio of 1:10 using a magnetic stirrer and holding at 80 °C for 1 h. The sample was then filtered through 0.7 µm glass microfiber filter paper (Whatman GF/F, Fisher Scientific, Loughborough, UK), the ethanol evaporated via rotary evaporation at 40 °C and the filtrate containing the lignin, and other ethanol-soluble components of the cocoa shell powder, recovered. The material, termed hydrothermal ethanol extract (HEE), was kept at 25 °C until further use.

Cocoa shell is known to contain metals. Therefore, the metal content of HEE, as used for colloidal particle preparation, was analyzed by Reading Scientific Sciences Ltd. (Reading, UK) using a 7700× ICP-MS System (Agilent, CA, USA) and an un-disclosed method. The levels were not of concern in view to future application of the colloidal particles as functional ingredient in processed foods.

#### Extracting Lipid

Hexane was used as a solvent to remove co-extracted lipids from HEE by heating 1:10 hexane:HEE for 2 h at 100 °C while continually agitating at 600 rpm on a magnetic stirrer. Following lipid solubilization, the sample was filtered through 0.7 µm filter paper. The extract was collected and dried to obtain the lipids removed hydrothermal ethanol extract (LR-HEE). The hexane contained in the filtrate was evaporated utilizing a rotary evaporator to obtain the extracted lipid.

### 2.6. Colloidal Particle Preparation

Colloidal particles were prepared via antisolvent precipitation adapting a method reported in [21]. HEE and LR-HEE respectively was re-dissolved into ethanol at a concentration of 2, 5 and 10 mg HEE/mL ethanol and 2 mg LR-HEE/mL ethanol while slowly stirring at 30 °C for 30 min on a magnetic stirrer. Each of the dilute extract solutions was then slowly poured into the vortex of water being agitated at 1000 rpm and left to stir for 24 h at 20 °C. Extract solution:water ratio was 3:7 and in each case, a total volume of 100 mL was processed in a 250 mL tall glass beaker (55 mm diameter, 120 mm high) using a cylindrical magnetic stirrer (5 mm diameter, 30 mm long). The colloidal particle dispersions prepared from solutions of 5 and 10 mg HEE/mL ethanol were diluted to have the same extract concentration as 2 mg HEE/mL prior to analysis. This step was undertaken to remove extract concentration as a factor potentially impacting the results.

Another process of droplet formation was applied to the 2 mg HEE/mL ethanol solution to assess the impact of droplet break-up and coalescence, likely to occur in the stirred process, on particle size. This process comprised dispensing the diluted extract directly into water, without agitation, using a syringe pump (Harvard Apparatus 22). The pump was fitted with stainless steel tubing (0.5 mm diameter) and set to dispense at 10 mL/h. The ethanol was left to evaporate over 3 days at 20 °C. All of the prepared colloidal particle suspensions were stored at 5 °C until further use.

### 2.7. Colloidal Particle Size and Zeta-Potential Methods

The particle size distribution and the zeta(ζ)-potential of the particles was analyzed in two independent measurements using the dynamic light scattering (DLS) and the electrophoretic mobility set-up with Smoluchowski model, respectively, on the Zeta Sizer (Malvern Zetasizer Nano ZS, Malvern Panalytical, Malvern, UK) [25]. For DLS measurements, a refractive index of 1.6 [26] and an absorption of 0.01 were used as diffraction pattern analysis parameters.

For sizing, the colloidal particle dispersions were placed, without further dilution, into disposable cuvettes and analyzed 1 day, 20 days and 100 days after production. The results are shown as the average of two sample sets with 15 runs. The large number of runs was chosen to validate that particle sedimentation was absent, which might have affected the results, reported as the particle diameter at peak intensity. The ζ-potential of the colloidal particle dispersions was analyzed 1 day after preparation in a folded capillary cell (Malvern, Zeta sizer nano series DTS1070, Malvern Panalytical, Malvern, UK).

### 2.8. Interfacial Property Methods

A profile analysis tensiometer (PAT1, Sinterface, Berlin, Germany) was used for contact angle, surface tension and interfacial tension measurements as follows. Contact angle was analyzed as a measure of the wettability of HEE and LR-HEE. Because HEE was paste-like whereas LR-HEE was of powder form, different methods of sample preparation for contact angle measurements were applied.

HEE was added into the bottom of the standard cubic glass cuvette of the equipment (20 mm) in sufficient quantity to cover the base, placed in a 40 °C vacuum oven (Weiss Gallenkamp, Leicestershire, UK) to aid spreading of the extract across the surface, and finally smoothed using an aluminum spatula. The extract was allowed to cool for 1 h before the cuvette was filled to the rim with sunflower oil. A 10 µL water droplet was then dosed using a Gilson pipette and allowed to sediment onto the extract surface for contact angle measurement. To closer reproduce the conditions under which the surface of the colloidal particles was formed, another sample was prepared by dissolving HEE (0.2 g) first in ethanol (0.1 mL) before placing into the cuvette. This mixture spread easily on the bottom surface of the glass cuvette. It was immediately covered with water and left at 20 °C for 24 h to allow ethanol diffusion into the water phase and evaporation. The water was then poured off the extract. As before, sunflower oil was added, a 10 µL water droplet pipetted into the cuvette and allowed to sediment to the surface of the extract before recording the contact angle.

The powdery LR-HEE was spread with a spatula onto sticky tape affixed to a glass slide cut into a 15 mm × 15 mm square (Silverline Diamond Glass Cutter, Somerset, UK). The spatula was gently tapped for even coverage. This was repeated 3 times to ensure full coverage before placing the piece of glass slide into the tensiometer cuvette, filling the cuvette with sunflower oil and pipetting a 10 µL water droplet onto the surface as before. Due to limited LR-HEE sample volume, no additional procedure for contact angle measurement, mirroring more realistically the particle surface properties as for HEE, was developed.

For all measurements, the shape of the water droplets was captured 30 s after dosing, using the camera set-up and frame grabber of the tensiometer. The images were then opened with a public domain image analysis software package (Image J), the asymptotes limiting the contact angle through the water phase manually applied and, finally, the contact angle value computed. An average of 10 measurements of left and right contact angle each are reported with each extract surface analyzed with two separate droplets. The described contact angle method was developed as the colloidal particles were in a water phase and too small to be applied to other contact angle measurement methods reported for Pickering particles, for example the gel trapping technique [27].

For surface and interfacial tension measurement, the tensiometer was fitted with a straight stainless-steel capillary (2 mm outer diameter) and a pendant drop of 26 mm^3^ constant volume was suspended into air and sunflower oil, respectively. Interfacial tension measurement was applied to the colloidal particle dispersions and, as aforementioned, to the oil phase following removal of surface-active molecules. Measurement temperature was 20 °C and equilibrium was reached after 1 h and 2 h, respectively. Results are reported as the average of the final value recorded for three runs, full data sets are depicted in Figure S1.

### 2.9. Emulsion Methods

Emulsions were prepared by emulsifying 1 g of sunflower oil into 9 g of each of the colloidal particle dispersions, contained in a small glass vial, with a high shear overhead mixer (T 18 digital Ultra-Turrax, IKA, Oxford, UK) operating at 9000 rpm for 2 min. Finally, the emulsions were stored in their glass vial at 5 °C. The microstructure of the emulsions and its evolution over storage of up to 7 days was then inspected visually using optical microscopy, as earlier described, and quantified by measuring the droplet size distributions using a low angle laser diffraction particle size analyzer (LS13 320, Beckman Coulter, High Wycombe, UK) fitted with an aqueous dispersion cell (Universal liquid module, LS12 320, Beckman Coulter, High Wycombe, UK). Three independent replicates of each sample were taken and data was analyzed using the Fraunhofer diffraction model within the equipment software. Results are reported as surface area-based density distributions of an average of three measurements on two sample sets acquired 1 day and 7 days after emulsion preparation.

### 2.10. Statistical Analysis

Statistically significant difference was assessed between two sample sets for contact angle measurements using the unpaired two tailed *t*-test. For colloidal particle size, ζ-potential of colloidal particles, surface tension and interfacial tension analysis one-way ANOVA and Tukey’s statistical test was carried out. For all analyzes the level of significance was set at *p* = 0.05.

## 3. Results and Discussion

### 3.1. Properties of the Cocoa Shell and Its Extracts

#### 3.1.1. Lignin Content, Solids Morphology and Chemical Fingerprint

**Figure 1:**
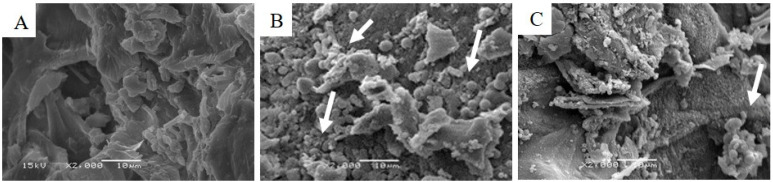
SEM micrographs of milled cocoa shell (**A**), milled cocoa shell particles after hydrothermal treatment (250 °C for 1 h) (**B**), and following ethanol extraction (**C**). The arrows in (**B**) point out surface-adhered droplets composed of lignin and lipids. Ethanol extraction on the hydrothermally treated solids has removed the majority of the surface-adhered droplets (**C**). The scale bars represent 10 µm.

The lignin content of the milled cocoa shell, assessed via the acetyl bromide assay, prior to and following hydrothermal treatment was 11.96 ± 0.70% and 28.72 ± 0.27%, respectively. The apparent increase in lignin content can be attributed to the easier accessibility of the assay’s solvent to the lignin following its at least partial relocation to the surface of the biomass particles during the hydrothermal treatment [19]. Evidence that the selected process conditions (250 °C, 1 h) applied to the milled cocoa shell feedstock led to this relocation is provided in Figure 1 by means of scanning electron micrographs. Figure 1A shows the surface of the untreated biomass. It appears relatively smooth and does not feature any of the distinct spherical surface adhesions which are clearly identifiable in Figure 1B, taken after the hydrothermal treatment. Their appearance and approximate diameter of a few microns is not dissimilar to those previously shown for hydrothermally treated coffee particles [11], corn stover [28], woody biomass [29], and corn cobs [30]. Figure 1C then shows the cocoa shell surface following ethanol extraction, applied to recover the lignin, and co-extracting other ethanol soluble components, as feedstock for the antisolvent precipitation of colloidal particles. It is clearly recognizable that the majority of the droplets have been removed in the extraction process. Analyzing the remaining solids for lignin content with the acetyl bromide assay revealed that they still contained 19.10 ± 0.02% of lignin. This suggests that there is opportunity for increasing lignin yield through using complementing pre-treatments, for example acid assisted hydrolysis [31].

**Figure 2:**
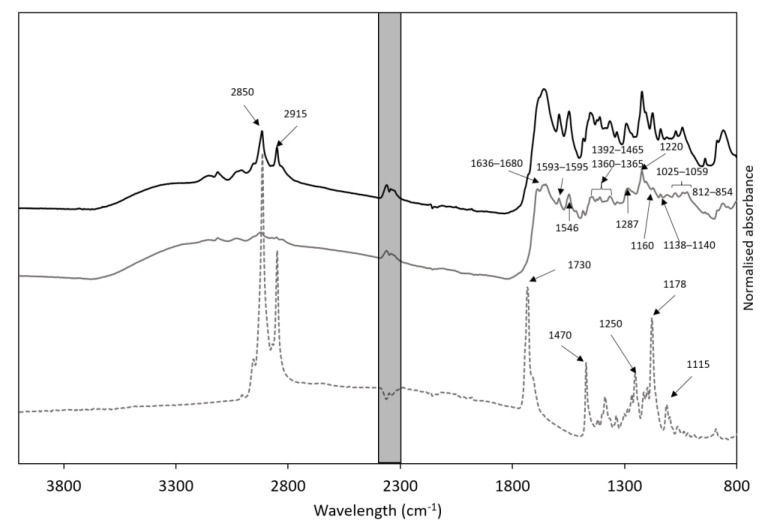
Normalized FT-IR profiles of HEE (hydrothermal ethanol extract, black line), LR-HEE (lipid removed HEE, grey line) and the extracted lipids (dashed line) in the range 800–4000 cm^−1^. Wavenumbers for the key peaks are highlighted, peaks at 2358 and 2340 cm^−1^ are related to the atmospheric conditions during measurement (greyed out area).

The acetyl bromide soluble (ABS) lignin content of the hydrothermal ethanol extract (HEE) was 47.14 ± 0.85% and via hexane extraction a lipid content of 53.06 ± 4.26% was found. The ABS lignin content of the lipids removed hydrothermal ethanol extract (LR-HEE) was 94.51 ± 4.84%. To further characterize the chemical composition of both extracts the FT-IR spectra depicted in Figure 2 were acquired. The third FT-IR data trace in Figure 2 refers to lipids extracted from HEE.

As expected, the spectra for HEE and LR-HEE show characteristic lignocellulosic peaks in the region of 800–2000 cm^−1^ [23]. The peaks at wavenumbers 1025–1059 cm^−1^ correspond to the O–H and the C–O stretch in cellulose, hemicellulose and lignin as well as the C–O aromatic ring and C–H primary alcohol found in lignin [32,33,34,35]. The peaks at 1160, 1410, 1465, 1636 and 1680 cm^−1^ relate to the C–O–C asymmetric stretching in cellulose, the C–H bond in polysaccharides, the C=O stretching found in lignin and the presence of conjugated carbonyl-carboxyl groups [23,36,37,38,39]. They also show a peak at 1660 cm^−1^ which relates to C=O found in lignin [40]. The peaks at 1360–1365 cm^−1^ and 1546 cm^−1^ relate to protein and are both seen in the HEE and LR-HEE spectra [41,42].

The spectra also show peaks relating to specific units of lignin; guaiacyl (G), syringyl (S) and p-hydroxylphenol (H) lignin [43]. The peaks at 1595 and 835 cm^−1^ have been reported to relate the S unit [40,44,45], the peak at 1663 cm^−1^ to the C=O conjugated p-substituent in the H unit [43] and the peaks at 1287, 1138-1140 and 854 cm^−1^ to the G unit [35,43,46,47]. Peaks at 1033 and 1220 cm^−1^ relate to both the S and G units [43,46,47,48,49]. These peaks were identified in both lignin-rich samples analyzed with the FTIR which suggests that their lignin composition was not different. This finding could be expected because treatment conditions affecting the lignin component were not varied in this study. However, one of these two samples had lipids removed and expectedly, there were differences in the lipid regions of the spectra as follows. The spectrum for HEE had two additional distinct peaks at 2850 and 2915 cm^−1^, identifying presence of triglycerides (TAG) and phospholipids, respectively [50]. Following hexane extraction, these lipid peaks were less pronounced and, as expected, they were present in the spectrum acquired on the extracted lipid. The additional peak at 1730 cm^−1^ corresponds to lipid esters [51,52,53]. Further peaks present in the extracted lipid spectrum include 1470, 1250, 1178 and 1115 cm^−1^, corresponding to CH_2_ in phospholipid [54], phospholipids [55], C–O stretch [56] and C–O vibration [57].

#### 3.1.2. Wettability

The wetting properties of HEE and LR-HEE were assessed via contact angle measurement to predict whether particles prepared from either extract would be predominantly hydrophobic or hydrophilic and, therefore, preferably stabilize w/o or o/w emulsions. Two different methods were applied as HEE was a paste-like material whereas LR-HEE was a dry powder.

In the case of the HEE, the contact angle of a sessile water droplet (10 µL) on the flattened extract surface confined in a cuvette filled with sunflower oil was assessed; example images are depicted in Figure S2. The average of 10 contact angle measurements (left and right) as measured via image analysis through the water phase was 98 ± 5°. This value is close to 90° suggesting that HEE would not be preferentially wetted by either oil or water [3]. As elaborated in the methods section, the wetting behavior of HEE was also examined following a sample preparation method designed to replicate the solvent conditions during particle formation. The average contact angle in this instance was 75 ± 7°. This value is significantly lower than before the pre-treatment of the extract surface (*p* = 0.05) and suggests that the CLRPs would be wetted by water and stabilize o/w emulsions [3]. The difference in value due to the treatment suggests that water may have been adsorbed by the extract, increasing hydrophilicity. This has been seen previously in clay particles having lowered contact angles when in contact with a water droplet before oil was added as the continuous phase to assess contact angle [58].

In the case of LR-HEE, the surface for contact angle assessment corresponded to a dry powder surface. The contact angle of a sessile water droplet placed onto the surface following coverage with sunflower oil was 123 ± 5°. The extract is clearly hydrophobic and it appears that the higher hydrophilicity of HEE was due to the presence of lipids, removed in the case of LR-HEE. However, LR-HEE and HEE were in different physical states, powder and paste, respectively, and, therefore, comparison should be taken with caution. A rough surface, as present in the powder case, has previously been found to repel water and oil from the surface of titanium dioxide nanoparticles compared to unmodified smooth surfaces [59]. Additionally, the angles measured for LR-HEE should be interpreted as apparent contact angle as the surface roughness may cause an overestimation due to possible air pockets remaining between droplet and surface [60].

### 3.2. Fabricated Colloidal Lignin-Rich Particles

The hydrothermal ethanol extract (HEE) and the lipids removed HEE (LR-HEE) were processed into CLRPs through antisolvent processing of their ethanol solution in water. Three solution concentrations (2, 5 and 10 mg HEE/mL ethanol) and two antisolvent process conditions (stirred versus nonstirred) for the lowest solution concentration were assessed for HEE. LR-HEE was precipitated into particles from 2 mg LR-HEE/mL ethanol concentration into water under constant agitation.

The obtained aqueous particle suspensions were assessed for ζ-potential and interfacial tension 1 day after processing. Their stability during storage at 5 °C was followed over 100 days by assessing particle size distribution on day 1, day 20 and day 100 using DLS. Additionally, individual particle morphology was assessed by acquiring TEM images, following drying of a sample of the particle suspension on TEM grids on day one.

#### 3.2.1. Morphology and Size

**Figure 3:**
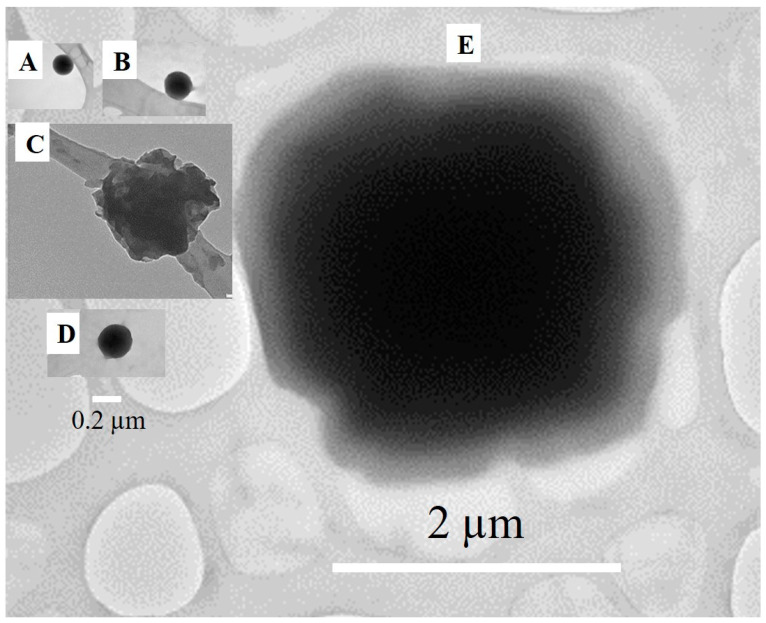
TEM image of individual colloidal particles (dark shape, the grey features are the TEM grid) produced by antisolvent precipitation into water of 2 (**A**), 5 (**B**) and 10 mg HEE/mL ethanol (**C**) on the magnetic stirrer, of 2 mg LR-HEE/mL ethanol on the magnetic stirrer (**D**) and 2 mg HEE/mL ethanol in nonstirred conditions (**E**). Images are scaled to align scale bars, representing 0.2 (**A**–**D**) or 2 µm (**E**).

Figure 3 shows TEM images of one individual particle for each of the particle preparation conditions. It was challenging to capture a particle in the field of view due to the very dilute nature of the particle suspensions. Hence, we discuss these images with caution. Figure 3A,B,D show that smooth and spherical particles were obtained from 2 mg and 5 mg HEE/mL ethanol, as well as 2 mg LR-HEE/mL ethanol precipitated in stirred conditions. The particle shown in Figure 3C for the 10 mg HEE/mL ethanol processed in stirred conditions appears partly translucent and angular. Unfortunately, the particle shown in Figure 3E, resulting from processing 2 mg HEE/mL ethanol in nonstirred conditions, is out of focus and its morphology could be either of the two observed.

**Figure 4:**
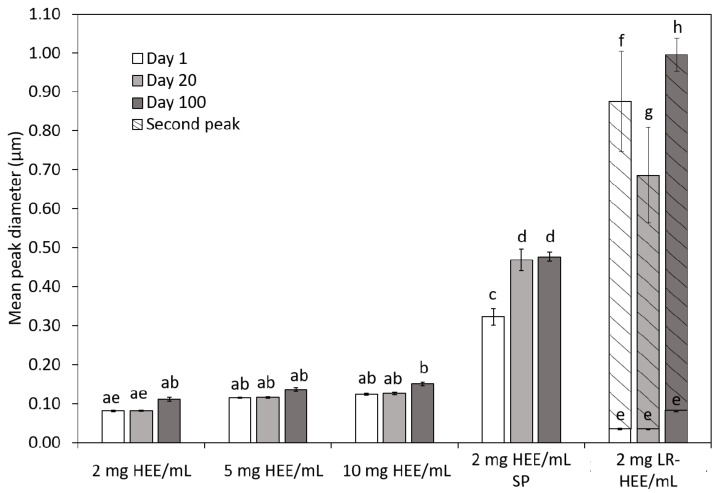
Mean peak diameter on day 1, day 20 and day 100 for the particles suspensions from 2, 5 and 10 mg HEE/mL magnetically stirred and 2 mg HEE/mL processed with syringe pump (SP) and from the 2 mg LR-HEE/mL ethanol magnetically stirred colloidal particle dispersions. Mean peak diameters relate to mean individual particle peaks, omitting aggregation peaks at 3.5–5.6 µm as explained in the main body text. LR-HEE showed a smaller and a larger mean individual particle peak identified by solid and dashed bars respectively. Different letters indicate statistically significantly different diameters, *p* = 0.05, ANOVA.

The results of the DLS measurements 1, 20 and 100 days following antisolvent processing are summarized in Figure 4. Presented are the mean peak diameters of the single particle populations, omitting the values for particle aggregates ranging between 3.5–5.6 µm. The fact that this larger peak, noticed for each sample as of day one except for 2 mg HEE/mL (agitated process) where it was only seen as of day 20, was due to aggregation was ascertained by applying ultrasound to separate the particles prior to analysis. Aggregation could be expected because the antisolvent phase was void of a stabilizing surface-active or polymeric additive. Particles produced from HEE under stirred conditions were all of similar mean peak diameter, around 0.1 µm. While not significant, mean peak diameter increased with increasing extract concentration and also over the 100-day storage period. Much larger particles were produced by changing the production process to using a syringe pump. The suspension was less stable as the mean peak particle diameter increased significantly over time. Production of larger particles is most likely due to the absence of flow stresses leading to droplet break up, as has previously been reported for stirred processes at low agitation intensity [61,62,63].

The resulting particle suspension from 2 mg LR-HEE/mL had two individual particle population peaks, with the first peak of 0.04 µm on day 1 and 20 and 0.08 µm on day 100 being statistically significantly similar to 2 mg HEE/mL following 1 and 20 days of storage (Figure 4). The second population peak diameter was statistically significantly larger and at 0.88, 0.68 and 0.99 µm following 1, 20 and 100 days of storage. Aggregation of LR-HEE particles was much more pronounced than for HEE particles. Hence, although not completely, the presence of lipids in the extract appears to have suppressed particle aggregation. Surface active fatty acids are commonly used as additives in colloidal particle production in the pharmaceutical industry [64,65]. The phospholipids coat the particles and prevent aggregation. The absence of surface-active lipids may also be the reason for the larger of the two individual particle populations. Their presence would decrease interfacial tension thus promoting droplet breakup and mixing of the solvent and antisolvent phase, a factor established to promote a small narrow antisolvent particle population [66]. It could also be that a fraction of initially smaller particles has coalesced into larger individual particles, a hypothesis requiring experimental validation.

The particle sizes obtained in this study are comparable to those reported in [21] on which we based our choice of precursor concentration in the solvent phase. Because they employed a dialysis tubing based antisolvent process, it is worth comparing our results to [67] where a stirred set-up similar to ours was used. Initially, they obtained larger particles of around 25 µm in diameter from a much higher starting concentration of around 1.5 g/mL. Upon addition of a surfactant, sodium dodecyl sulphate, and varying temperature (20–60 °C) they then could produce smaller particles (0.1–0.2 µm). The present study has shown that surfactant contained in the extract can play the same role. This is an advantage in the context of clean label products and maximization of resource utilization as one could imagine to increase the level of indigenous surface-active components in the extract through purposely designed extraction regimes. These co-extracts may possess additional benefit as natural antioxidants [68].

#### 3.2.2. Zeta Potential (ζ)

ζ-potential was analyzed to further assess the particle stability, at a pH of 5.96 ± 0.58 for all samples. Table 1 shows that the ζ-potential of the HEE particle suspensions produced via magnetic stirring was around −45 mV. Removing lipids prior to particle precipitation led to a lower ζ-potential compared to the HEE particles prepared by magnetic stirring but the largest ζ-potential was from the syringe pump method, suggesting the size of the particles influences the ζ-potential. Despite significant differences between values as indicated in Table 1, the values are close to each other compared to the value range reported in literature. Ζ-potential values of around −40 mV have been found for other lignin microparticles [69] and Lievonen et al. (2016) reported ζ-potential values of up to −60 mV for their lignin nanoparticle suspensions [21]. Generally, it is considered that particles with a ζ-potential in the range of ±30 mV are stable [70]. The ζ-potential data reported here was taken on day one and suggests no aggregation should have occurred. 

#### 3.2.3. Surface Activity at Air/Water and Water/Oil Interface

Table 2 reveals that the presence of particles in water decreased the surface tension at the air/water interface from 72 mN·m^−1^ to around 40 mN·m^−1^ for particles prepared from HEE and to 53 mN·m^−1^ following lipid removal. At the oil-water interface all suspensions reduced the interfacial tension from 27 mN·m^−1^ to around 12–14 mN·m^−1^. Removal of lipids led to statistically significantly higher values for surface and interfacial tension (Table 2). This is not surprising as lignin and lipids are both known to be surface active [71,72,73], although the above discussion on particle size and aggregation highlighted the critical role surface-active lipids, and not lignin, appear to play in the system studied.

Depending on the initial concentration of HEE in ethanol and the colloidal particle production process the surface tension values differed. The largest surface activity was seen for suspensions with larger particles (Figure 4), possibly from an increase in coverage of the oil/water interface. Similar findings have been reported in [74] with increasing volume fraction of particles causing an increase of interface coverage and reduction in surface tension. It should be noted though that these authors kept the size of the particles constant, so a direct comparison cannot be made.

In terms of interfacial tension, all suspensions reduced the interfacial tension at the water/oil interface compared to the bare water/oil interface. The resulting values vary very little, but it is noteworthy that the particles produced in the presence of lipids reduced the interfacial tension to a significantly lower value than the particles produced after lipid removal at otherwise the same conditions. This result validates either the surface presence of lipids at the CLRPs or the presence of lipids in the aqueous phase of the particle suspension. Further experimental validation is required, also bearing in mind that LR-HEE had a slightly smaller and larger population compared to the monomodal individual particle peak of HEE.

### 3.3. Emulsion Stabilization

The CLRP suspensions were assessed for their ability to stabilize in o/w Pickering emulsions. Micrographs and droplet size distributions were acquired 1 day and 7 days after producing 10% oil-in-water emulsions via high shear mixing.

**Figure 5:**
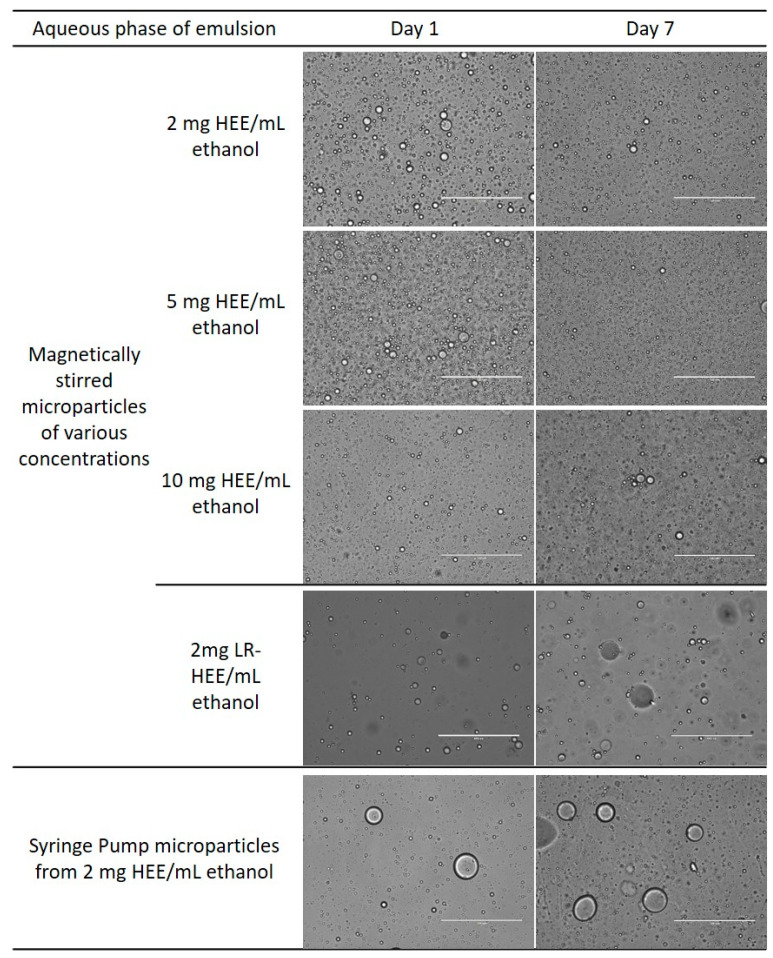
Microstructure of o/w emulsions 1 and 7 days post production. Scale bars represent 100 µm.

The micrographs in Figure 5 show that the particles produced via magnetic stirring produced emulsions with a large number of small oil droplets (0.5–15 µm) which were slightly flocculated. Using a syringe pump to produce the particles resulted in larger oil droplets (1–35 µm), with more of these seen on day seven. Removing the lipids resulted in emulsions with fewer small oil droplets and larger oil droplets seen over time, 1–4 µm on day one and 1–30 µm on day seven indicative of an emulsion unstable to coalescence, as confirmed by droplet size distribution data discussed later on. This instability observation for LR-HEE supports the idea that HEE particle suspensions contained surface-active lipids in their aqueous phase. This would lead to the initial stabilization of the processed emulsions, with long term stabilization imparted by the comparatively slower adsorption of the CLRPs at the emulsion interface. Indeed, fatty acids are known to stabilize o/w emulsions for a limited time when used below their critical concentration [75]. It has also been demonstrated previously that the presence of the fatty acids can enhance the functionality of Pickering particles, improving the stability to emulsion droplet coalescence [76]. It should also be noted that the contact angle for the lipid-removed extract was around 120° and therefore LR-HEE could be expected to favor the stabilization of w/o emulsions over o/w emulsions [3]. However, the oil:water ratio of 1:9 applied here would be highly unlikely to force phase inversion, but it might actually be seen at increased oil phase volume in future research.

**Figure 6:**
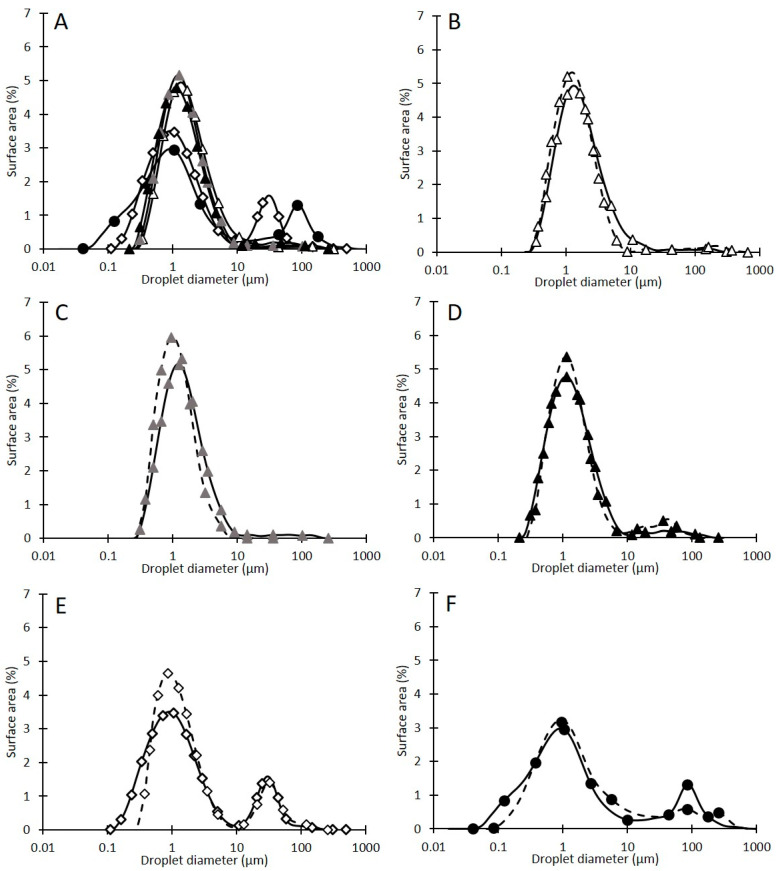
Surface area (%) distributions of the droplet size in the produced o/w emulsions on day one (**A**) from colloidal particle dispersions produced by antisolvent precipitation of lignin in an ethanol solution and water at concentrations of 2 (△) (**B**), 5 (▲) (**C**) and 10 mg HEE/mL ethanol (▲) (**D**) via magnetic stirring, 2 mg HEE/mL via a syringe pump method (¯) (**E**) and 2 mg LR-HEE/mL ethanol (●) (**F**) via magnetic stirring. Individual graphs represent oil droplet diameters following 1 day (solid line) and 7 days (dashed line) post production for colloidal particle dispersions.

The corresponding droplet size distributions for day one are depicted in Figure 6A. Figure 6B–F show the data by emulsion and include the data acquired on day seven. HEE particle stabilized emulsions had a main droplet size fraction in the range of 0.15–10 µm and a minute fraction between 10–100 µm. This fraction is significant though for the emulsion stabilized with the syringe pump produced HEE particle suspension, right from day one. The larger droplet sizes in this emulsion correlate to the larger size of the CLRPs [77,78]. The droplet size distribution for this emulsion also showed a slight shift at the lower end of droplet sizes to larger values of the storage period. This observation is in accordance with the coalescence identified on the micrographs (Figure 5). However, the distribution of the larger droplet fraction measured on day one and day seven overlap. It can be speculated that, and this would need to be validated through high resolution imaging of the emulsion interfaces, the droplets are initially not fully covered with particles and up until that is the case coalescence occurs.

When the LR-HEE particle suspension was employed as the continuous emulsion phase, the first droplet size peak was broader, and the secondary peak was shifted to the larger value of around 100 µm. This peak was less pronounced on day seven, which might be due a bulk oil phase appearing on top of the emulsion thus diminishing this fraction in the remaining emulsion phase. Clearly, as already identified through microscopic observation (Figure 5), this emulsion is not stable.

## 4. Conclusions

A lab-based process to produce colloidal lignin-rich particles with Pickering functionality from cocoa shell has been introduced. With the food industry as the supplier of the feedstock, it is anticipated that these particles will be accepted as food emulsion ingredients. Precipitating ethanol-dissolved lignin-rich cocoa shell extract into water spherical particles of a primary size range of 0.03–0.40 µm were obtained. Larger particles were obtained by dripping extract solution into non-agitated water, and smaller particles when lipids were removed from the extract. The particles reduced surface and interfacial tension with the larger particles lowering the surface tension to a greater extent, possibly due to an increased surface coverage. The particles formed from lipid-removed extract lowered the surface tension to a lesser extent, as expected from removing surface-active lipids such as phospholipids. The smaller size range of particles produced via the agitated process successfully stabilized 10% *w*/*w* sunflower o/w emulsions with good stability over seven days of storage. The larger particles produced emulsions that showed coalescence of small droplets. Particles processed from lipids removed extract failed to produce a stable sunflower o/w emulsion. It was concluded that the presence of indigenous lipids in the cocoa shell extract is a requirement to obtain particles with Pickering functionality.

The particles introduced here were prepared in aqueous suspensions. Therefore, their preferred application would be in water-based food. However, the contact angle of a water droplet placed on the cocoa shell extract was close to 90°, and around 120° for lipid-removed extract. Hence, there is potential for application in oil-based foods (w/o emulsions) provided the particles can be transferred into the oil phase for emulsion processing. Additionally, utilizing other lignin-rich co-products, which would inevitably be of different lignin unit composition, could potentially lead to more hydrophobic particles. Future research is required to understand the particle formation from the unrefined precursors applied here compared to most literature utilizing more or less pure lignin systems.

## Figures and Tables

**Table 1:** ζ-potential measurements for colloidal particle dispersions at pH 5.96 ± 0.58. Different letters indicate statistically significant differences, *p* = 0.05, ANOVA.

Extract	Process	Extract in Ethanol (mg/mL)	ζ-Potential (mV)
			Day 1
Hydrothermal ethanol extract (HEE)	Magnetically stirred	2	−44.88 ± 0.70 ^a^
		5	−45.87 ± 1.43 ^b^
		10	−44.74 ± 1.02 ^a^
	Syringe	2	−47.86 ± 1.28 ^c^
Lipid removed HEE	Magnetically stirred	2	−46.84 ± 0.75 ^d^

**Table 2:** Surface tension of colloidal particle dispersions from the hydrothermal ethanol extract (HEE) and lipids removed HEE (LR-HEE) from first peak, at various concentrations (2, 5, 10 mg/mL) and two production techniques (magnetic stirring and syringe pump). Different letters indicate statistically significantly different surface tensions with surface tension and interfacial tension assessed separately. *p* = 0.05, ANOVA.

	Process	Extract in Ethanol (mg/mL)	Surface Tension (mN·m^−1^) at 1 h	Interfacial Tension (mN·m^−1^) at 2 h
Hydrothermal ethanol extract (HEE)	Magnetically stirred	2	42.6 ± 1.1 ^a^	12.4 ± 1.2 ^a^
		5	40.9 ± 1.1 ^ab^	13.2 ± 0.7 ^ab^
		10	38.8 ± 1.2 ^b^	12.1 ± 0.3 ^a^
	Syringe pump	2	36.5 ± 3.3 ^b^	14.0 ± 1.2 ^b^
Lipid removed HEE	Magnetically stirred	2	53.0 ± 1.5 ^c^	14.4 ± 0.3 ^b^

## Data Availability

Not applicable.

## Supplementary Materials

The following are available online at https://www.mdpi.com/2304-8158/10/2/371/s1, Figure S1: Interfacial tension data of microparticle suspensions; Figure S2: Contact angle measurements of hydrothermal ethanol extract (left and middle) and lipid removed hydrothermal ethanol extract (right).
