# Supplementary material for: Colloidal Particles for Pickering Emulsion Stabilization Prepared via Antisolvent Precipitation of Lignin-Rich Cocoa Shell Extract
Source: Foods. 2021 Feb 9;10(2):371. doi: 10.3390/foods10020371 (PMC7914710; doi:10.3390/foods10020371)
Supplement: Supplementary file 1 [file foods-10-00371-s001.pdf]

Figure S1. Interfacial tension data of microparticle suspensions; 2, 5, 10 mg HEE/mL and 2 mg LR-HEE/mL produced through magnetic stirring and 2 mg HEE/mL produced via syringe pump, against air (left) and sunflower oil (right). Note the different axis scales.

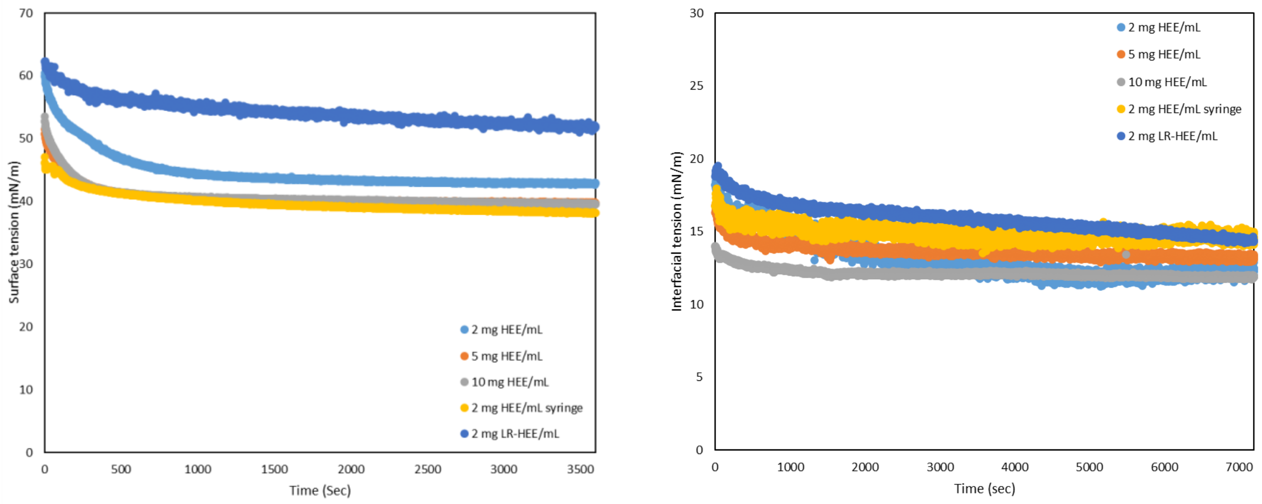

Figure S2. Contact angle measurements of hydrothermal ethanol extract (left and middle) and lipid removed hydrothermal ethanol extract (right). Angles were measured through the water phase.

Hydrothermal ethanol extract		Lipid removed hydrothermal ethanol extract
No Modification	Immersed in water	No modification
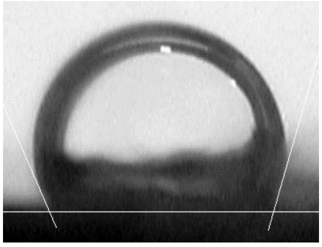	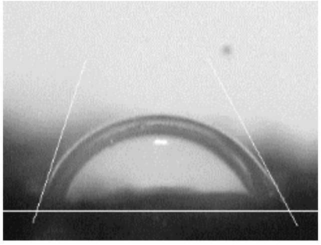	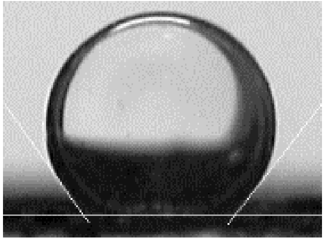
98 ± 5 °	75 ± 7 °	123 ± 5 °
